# A genetically enhanced sterile insect technique against the fruit fly, *Bactrocera dorsalis* (Hendel) by feeding adult double-stranded RNAs

**DOI:** 10.1038/s41598-017-04431-z

**Published:** 2017-06-22

**Authors:** Muhammad Waqar Ali, Wenping Zheng, Summar Sohail, Qingmei Li, Weiwei Zheng, Hongyu Zhang

**Affiliations:** Key Laboratory of Horticultural Plant Biology (MOE), State Key Laboratory of Agricultural Microbiology, Institute of Urban and Horticultural Entomology, College of Plant Science and Technology, Huazhong Agricultural University, Wuhan, 430070 China

## Abstract

RNAi based sterile insect technique (SIT) is an authentic insect management approach but requires proper target genes. During this study, spermless males were developed by interfering with germ cell differentiation and azoospermia related genes. Data demonstrates significant reductions in the target genes expressions (*boul*, *zpg*, *dsx* ^*M*^, *fzo* and *gas8*) after oral dsRNAs administration. Knock down of target genes significantly affected the reproductive ability of males and reduced egg-hatching as compared to the control group. Furthermore, different combinations of selected gene dsRNAs (*boul* + *zpg*, *boul* + *dsx* ^*M*^ and *zpg* + *dsx* ^*M*^) were made, which resulted up to 85.40% of male sterility. The most effective combination was selected to prepare different concentrations of dsRNA, 250, 500, 750 and 1000 ng/μl, that caused 18.97%, 38.68%, 58.02% and 85.40% male sterility, respectively. Subsequently, 1000 ng/μl of the same combination of ds-RNAs was used against differently aged adult flies (1, 5, 7, 10 days) which lead to 85.40%, 31.42%, 21.76% and 9.90% male sterility, respectively. SIT developed in this study showed that, *boul* + *zpg* combination of dsRNA feeding for 6 hours significantly reduced the number of spermatozoa and viability of sperm in 1-day-old *B. dorsalis* flies. In short, this study provides an effective SIT technique for long-term *B. dorsalis* management.

## Introduction

The oriental fruit fly, *Bactrocera dorsalis* (Hendel) is the notorious and most dangerous horticultural pest^1^. It attacks and destroys 250 different types of profitable fruit and vegetables on different continents of the world, especially Africa and Asia^2^. To control the fruit fly population by using the traditional pesticides is considered to be the best and quickest method. However, because of resistance to pesticides negative impact on human health and being dangerous for natural ecosystems, the development a new strategy to keep the pest population limited is needed^3^.

The sterile insect technique (SIT) is an environmentally friendly, biological and non-insecticidal tool to reduce the size of agricultural pest populations from the fields^4^. By releasing the infected or sterile males into the fields to mate with wild females, the next generation will be significantly reduced^5^. SIT is a very popular technique, especially for the control of the tephritid fruit fly, tsetse flies, screwworms and mosquitoes^6^. Currently, chemosterilants and radiation are often used to produce the sterilized male flies. Although radiation is quite effective, it is limited because of the need to install proper sources of radiation. In addition, the somatic damage to the insect caused by radiation inescapably reduces the competitive ability of male flies to mate with wild females^7, 8^. Loss of the ability of the males to reproduce ultimately reduces the pest population efficiency^3^. More recently, when traditional SIT had failed in the olive fruit fly due to an altered mating behavior in SIT-flies, genetically enhanced SIT showed great potential to control the pest population^3^. They also reported that *Bol* gene dsRNA treated insects have strong competiveness with the wild females and also caused suitable refractoriness to wild fruit fly females of olive^3^.

Detailed study by Ant *et al*., (2012) proved that weekly release of *Bol* treated males in wild female population resulted in sudden population down and can lead to eradication^3^. The production of sterilized male mosquitoes by using RNAi mediated knockdown of male fertility genes showed very good results after being released into wild populations^4^. Testis related target genes dsRNA harshly effected male reproduction of mosquitoes and produced up to 90% sterilized male population. In the same experiment Whyard *et al*., (2015) also observed the effect of female specific *double-sex* (*dsx* ^*f*^) and by sex determination they were able to produce the high number of male population by insects sex-sorting genes before the release^4^.

The change in spermatogenesis phases are controlled by dynamic gene expression. Many important signaling pathways, including EGFR and EGF signaling^9^, Activator of transcription (JAK-STAT) signaling and transducer^10^ and Bone Morphogenetic Protein (BMP) signaling^11^, exert transcriptional as well as post-transcriptional supervisory functions in spermatogenesis, and the JAK-STAT and BMP pathways perform are involved in the preservation of germline stem cells (GSCs)^11^. The *boule* gene showed defects in spermatid differentiation and played a very important role in coordinating the events of spermatid and meiosis^12^. During spermatocyte maturation, the *boule* gene plays an important role in generating dynamic *Cdk1*/*CycB* ^13^. In spermatogenesis, the development of the GSC occurs at a sex-specific stage to promote male dominant characteristics, which requires the male-specific sequence of the gene *double sex male* (*dsx* ^*M*^)^14^. *Zero growth Population* (*Zpg*) plays a very important role in producing spermless males^5^. The protein of *zpg* is located very close to the surface of spermatogonia, next to the somatic cyst cells^15^. Spermatogenesis oriented genes have the potential to induce sterility in adult males^16^.

In the present study our main objectives were to identify and clone the genes related to spermatogenesis in the model pest *B. dorsalis*. Screening of target genes and the combination of orally administered engineered-bacteria expressing dsRNAs of different genes can lead to male sterility, establishing a SIT technique in *B. dorsalis* using RNAi. It also confirms that RNAi is an alternative technique to radioactivity and it provides a new option for controlling other agricultural pest populations.

## Results

### Selection of testis specific genes

**Figure 1:**
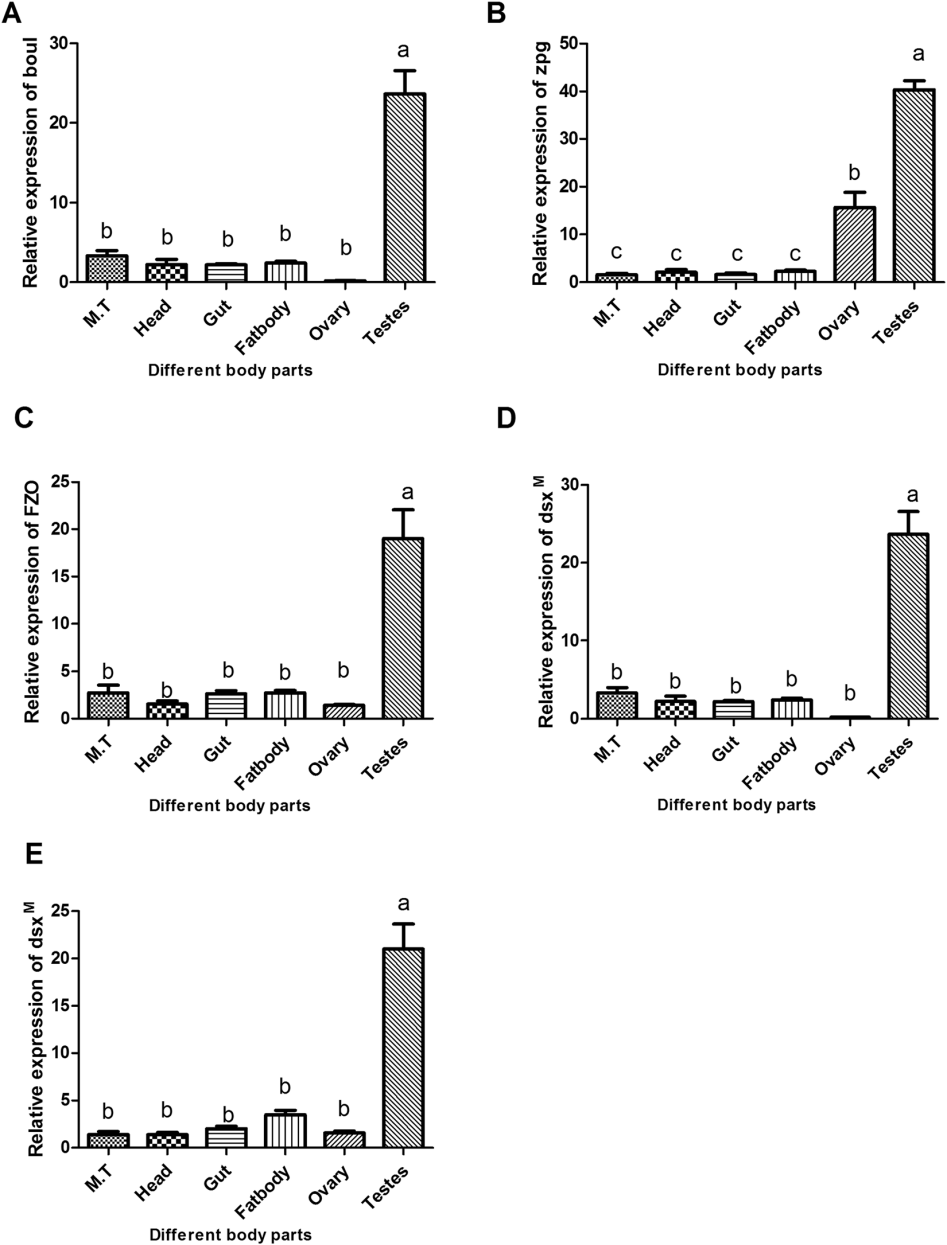
Differential gene expression in various body parts of *Bactrocera dorsalis* adults including testis, ovary, fat body, gut, head, and malpighian tubules, in response to selective genes (*Boul*, *Zpg*, *dsx* ^*M*^, *Fzo* and *Gas8*). Bars and different letters indicate significant differences in the expression level at p < 0.05 according to Tukey’s test.

To check whether the dsRNA of target genes will aid in developing the SIT technology in *B. dorsalis*, ten genes were selected based on previous studies^4^ and their homologous genes in *Drosophila melanogaster* were also identified (Table S1). Expression patterns of these genes in testis and ovary was analyzed by qRT-PCR using the primers (Table S2). Our results showed that 5 genes are highly expressed in the testis-specific of *B. dorsalis*, including *boul, zpg, gas8, fzo* and *dsx* ^*M*^ (Fig. 1) and these testis-specific expressed genes were selected for further investigation.

### Gene silence effects using RNAi

Based on the *B. dorsalis* transcriptomic data, RT-PCR was used to characterize the selected target genes using primers (Table S3). The partial nucleotide sequences of genes *boul*, *zpg*, *gas8*, *fzo* and *dsx* ^*M*^ were determined to be 905, 909, 1953, 1362 and 1959 bp, with having an ORF of 273, 725, 1434, 1164 and 1203 bp of amino acid, respectively. These sequences are highly conserved with high similarity with *D. melanogaster*.

**Figure 2:**
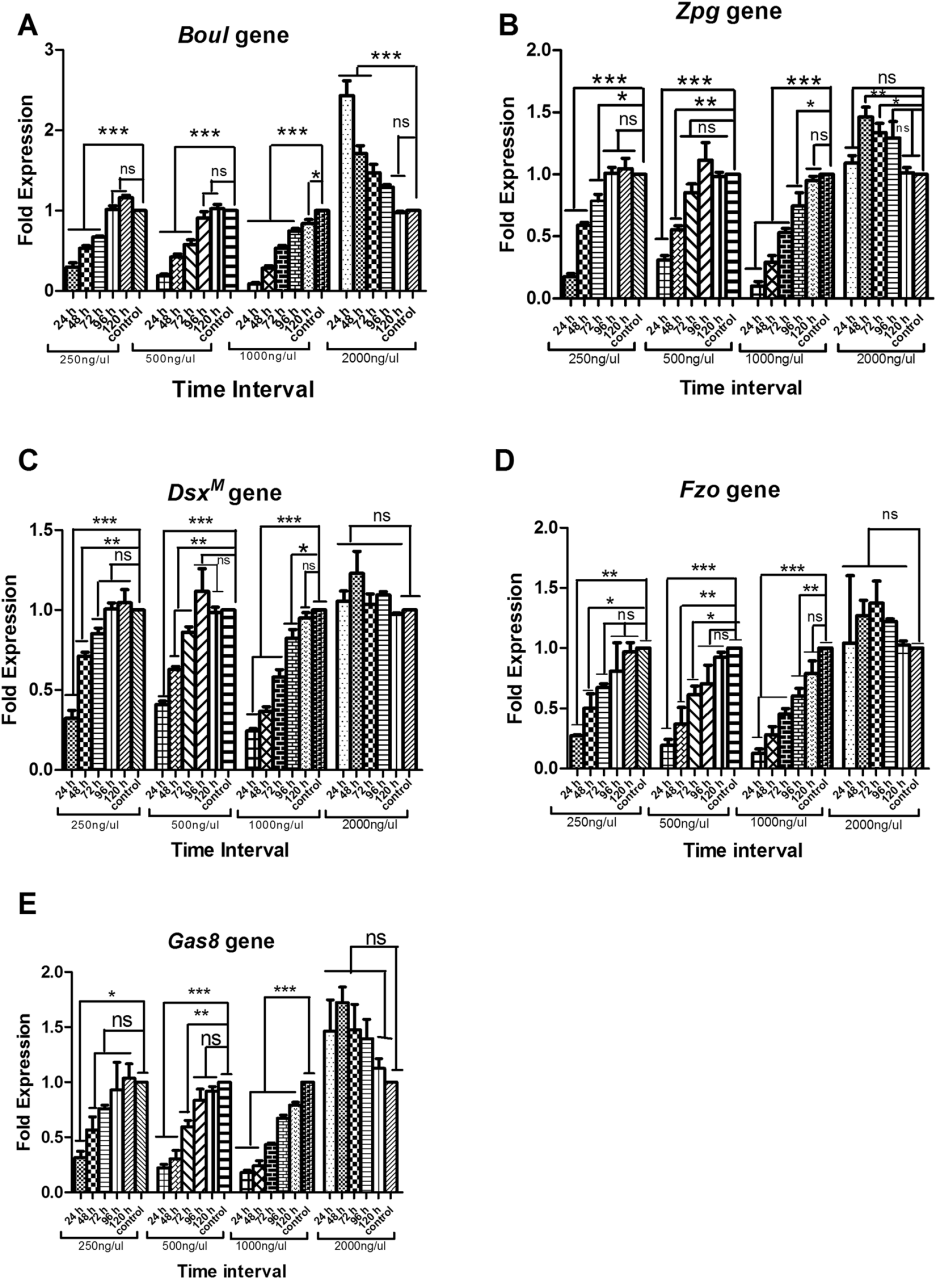
Effects of RNAi silencing of target genes on five consecutive days at different concentrations. Effect of orally administered dsRNAs against target genes (*boul*, *zpg*, *dsx* ^*M*^, *fzo* and *gas8*) (**A**–**E**) at different concentrations (250, 500, 1000 & 2000 ng/ul) were analyzed. Normalized target gene expression is reported relative to the expression of the ds-EGFP control, which was set to 1-fold. All error bars depict the SE of the mean of three independent replicates. Treatments were compared with their respective controls using ANOVA (Dunnett-test, P < 0.05). *, **, *** and ns indicates P < 0.05, P < 0.01, P < 0.001 and non-significant, respectively.

In response to different concentrations during the feeding of selected dsRNA, silencing effects on target genes were assessed for 5 consecutive days. According to reverse transcriptase scrutinizing revealed that maximum down regulation was noted at 1000 ng/μl concentration after 24 hours post exposure of dsRNA. The target genes *boul*, *zpg*, *dsx* ^*M*^, *fzo* and *gas8* showed 0.09, 0.10, 0.24, 0.13 and 0.18-fold decrease compared with 1-fold of ds-EGFP. After 48 hours of dsRNA feeding, the genes showed high expression as compared to 24 hours, with 0.28, 0.29, 0.37, 0.28 and 0.24-fold change in *boul*, *zpg*, *dsx* ^*M*^, *fzo* and *gas8* respectively (Fig. 2). The highest concentration (2000 ng/μl) showed interesting results. In response to 2000 ng/μl genetically engineered expressed dsRNA bacteria showed a significant increase in expression compared with 1-fold of ds-EGFP. After 24 hours of feeding target gene (*boul*, *zpg*, *dsx* ^*M*^, *fzo* and *gas8*) dsRNA at a concentration of 2000 ng/μl, the insects showed refractoriness of the dsRNA. The expression level of target genes was noted as 2.43, 1.09, 1.05, 1.04 and 1.46-fold, respectively, compared with 1-fold of ds-EGFP (P < 0.0001) (Fig. 2). Over time, the insect’s body tries to stabilize itself and gene expression returns to its normal condition. On 5^th^ day of treatment at 2000 ng/μl concentration the expression level was noted as 0.97, 1.01, 0.97, 1.03 and 1.13-fold in response to *boul*, *zpg*, *dsx* ^*M*^, *fzo* and *gas8* ds-RNAs, respectively.

### Screening of target genes for SIT based on male sterility

**Figure 3:**
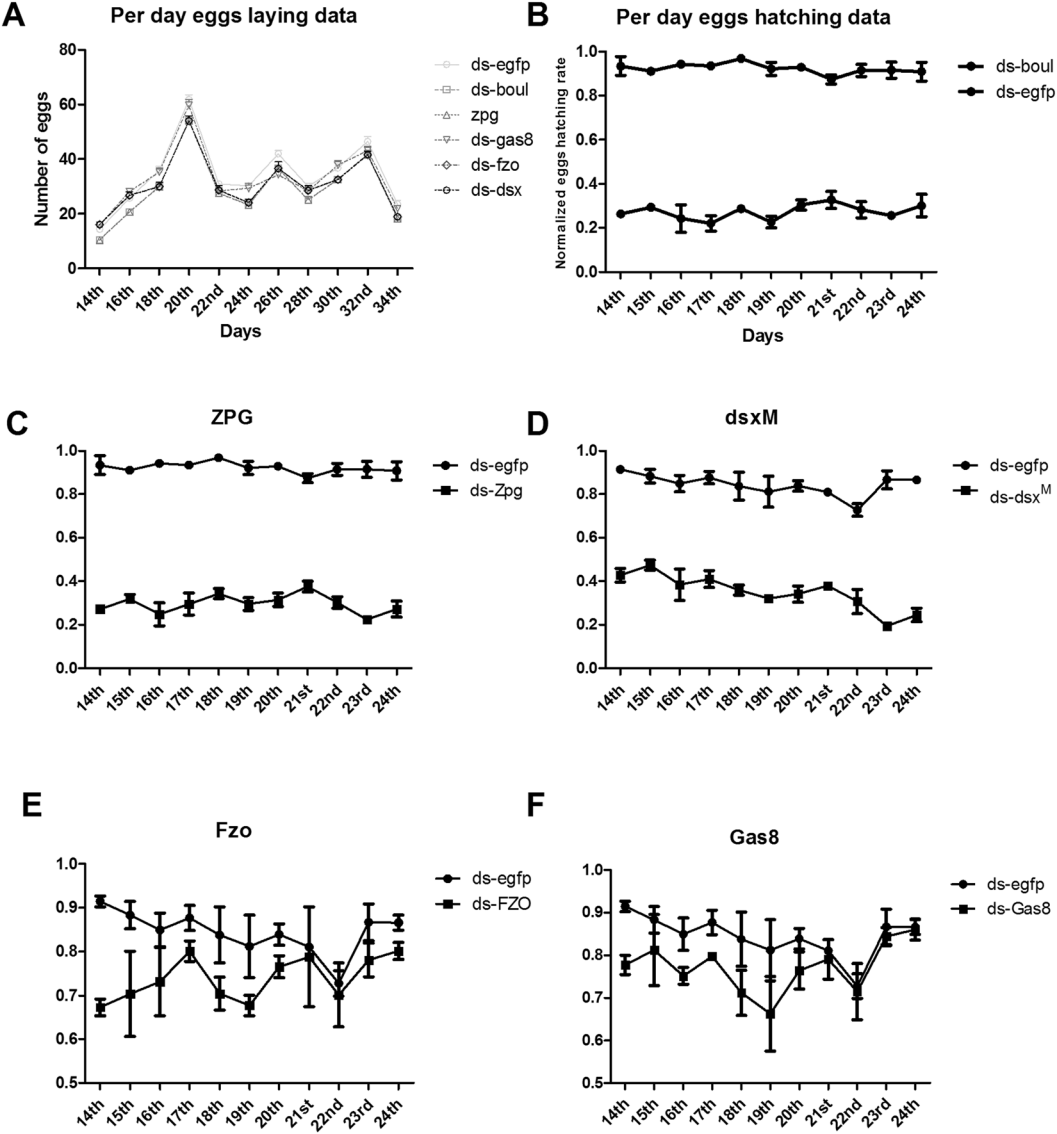
Egg laying and hatching capacity of *B. dorsalis* against dsRNA of target genes (b*oul*, z*pg*, *dsx* ^*M*^, f*zo* and g*as8*) with EGFP as a control.

To confirm whether these selected genes are suitable for SIT, daily numbers of laid eggs and the hatching rates were analyzed. The results showed that there is no significant difference in the numbers of laid eggs between the target gene dsRNA feeding and control (ds-EGFP) groups (Fig. 3A). Same testis-specific genes were selected and the effect of the dsRNA on male fertility was examined. The target genes showed a significant impact on the hatching rate of the eggs. Among the five genes, *boul*, *zpg* and *dsx* ^*M*^ showed 67.59%, 64.84% and 58.57% males sterility, respectively, while the *fzo* and *gas8* dsRNAs displayed only 19.96% and 16.40% reduction in egg hatching compared to the control group (ds-EGFP) (Fig. 3).

### Effects of a combination of different genes on male sterility

**Figure 4:**
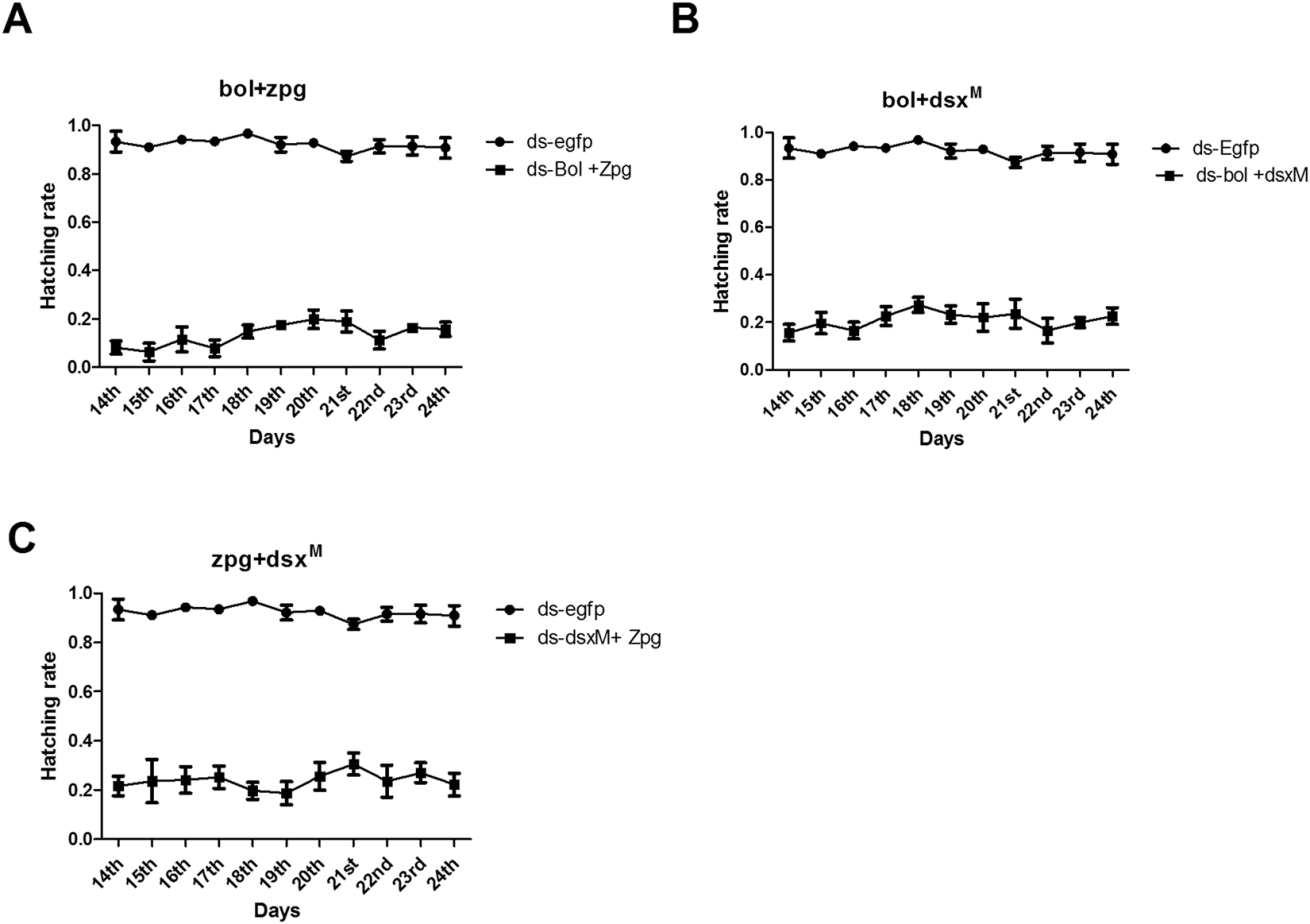
Egg hatching rate per day in response to a synergistic effect of two dsRNAs. The average number of eggs hatched per day between candidate gene dsRNA combinations and ds-EGFP treated flies (**A**) *boul* + *zpg* (**B**) *boul* + *dsx* ^*M*^ & (**C**) *zpg* + *dsx* ^*M*^). Three biological replicates were performed and significant differences in egg hatching rates were found.

**Figure 5:**
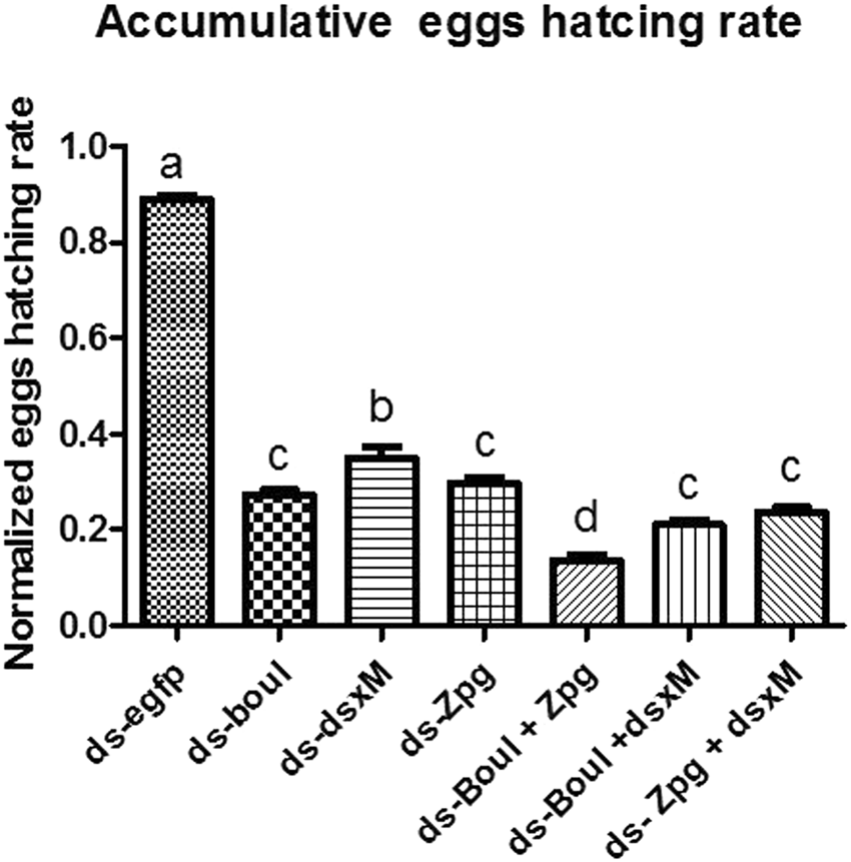
Egg hatching rate in response to individual and combinations of target gene dsRNA compared with ds-EGFP treated flies. Three biological replicates were performed, *** indicates significant difference. One way ANOVA indicate difference in average number of hatchings between all candidate genes dsRNA (P < 0.0001, Tukey test).

To enhance the sterility effect, target gene dsRNA combinations were made. We made three different combinations, *boul* + *zpg*, *boul* + *dsx* ^*M*^ and *zpg* + *dsx* ^*M*^. Based on the different combinations of orally administered dsRNA, hatching data were assessed in percentage. The synergistic effect of two different dsRNAs again showed egg laying results similar to feeding of single target gene dsRNA, while the impact on egg hatching rate showed strong and significant results. The cumulative reduced hatching rate in response to *boul* + *zpg*, *boul* + *dsx* ^*M*^ and *zpg* + *dsx* ^*M*^ were noted as 85.40%, 77.39% and 74.17% respectively compared with the control (Figs 4 and 5).

### Technique factors for SIT based on most effective dsRNA combination

**Figure 6:**
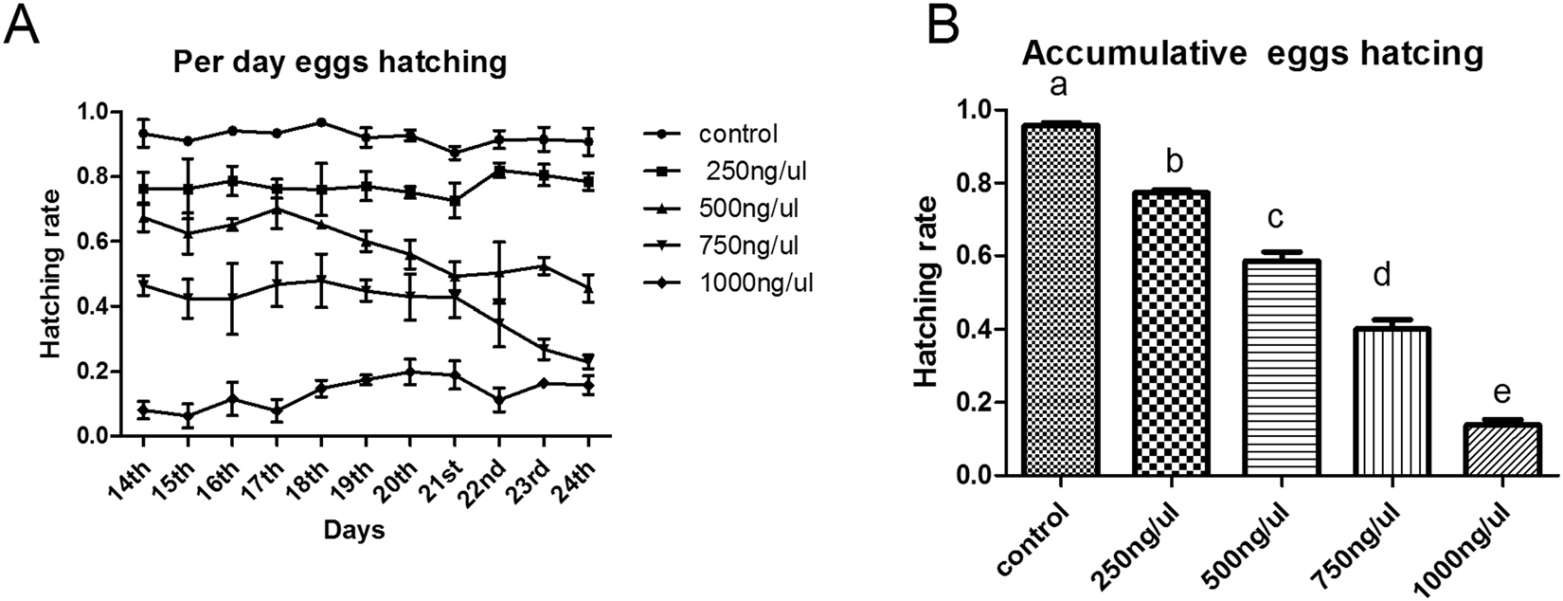
Egg hatching rate in response to different concentrations of dsRNA for the *boul* + *zpg* genes. (**A**) The average number of eggs hatched per day between target gene dsRNA and ds-EGFP at different concentrations. (**B**) One way ANOVA indicates a difference in the accumulative number of eggs hatched at different concentrations compared with the control group. Different letters indicate the significant differences between all treatments. Three biological replicates were performed (P < 0.0001, Tukey test).

According to the previous experiments, *boul* + *zpg* caused the highest degree of sterility among all of the tested dsRNAs. The synergistic effects of dsRNAs were again evaluated at different concentrations (250, 500 and 750 ng/μl) in newly emerged male. Young adults showed 18.97%, 38.68%, 58.02% and 85.40% (Fig. 6) sterility with 250 ng/μl, 500 ng/μl, 750 ng/μl and 1000 ng/μl, respectively compared with the control.

**Figure 7:**
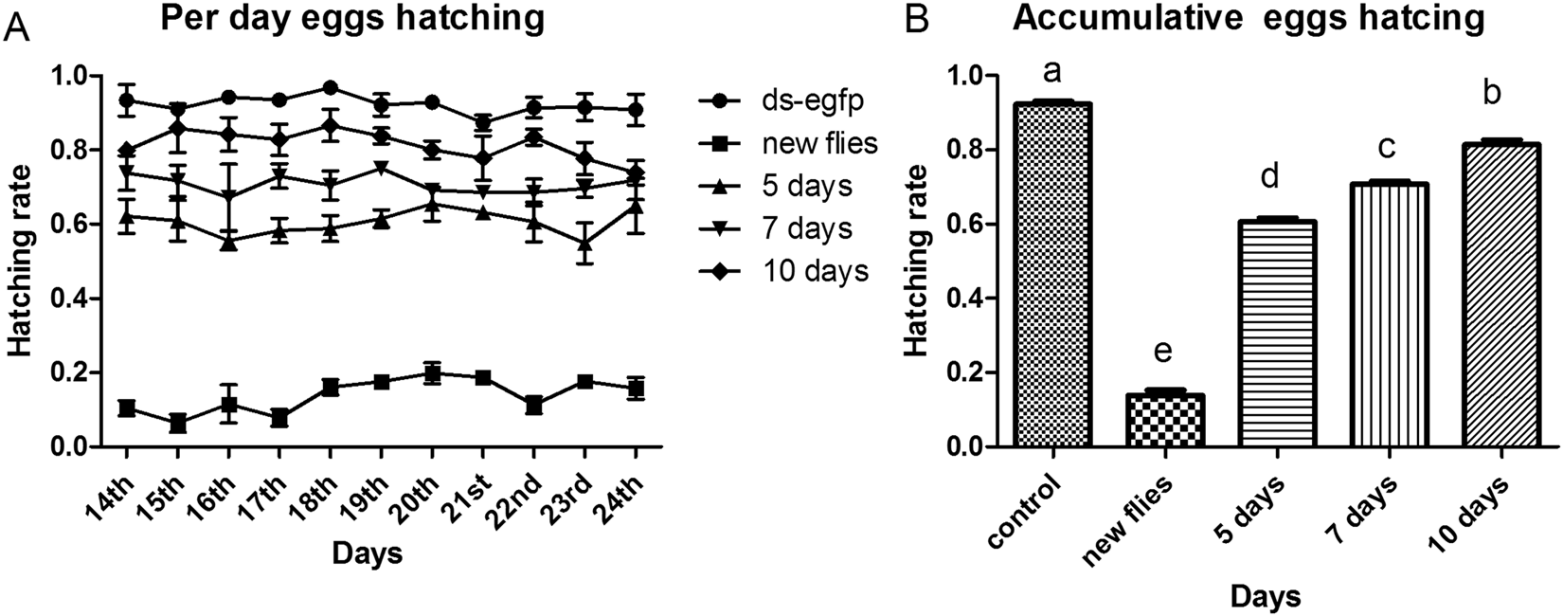
Egg hatching rate in response to 1000 ng/μl of dsRNA (*boul* + *zpg*) genes in different ages of adult males. (**A**) The average number of eggs hatched per day between target gene dsRNA and ds-EGFP at different concentrations were noted. (**B**) One way ANOVA indicates a difference in the accumulative number of eggs hatched after feeding 1000 ng/μl dsRNA to males of different ages at compared with the control group (ds-EGFP). Different letters indicated the significant differences between all treatments. Three biological replicates were performed (P < 0.0001, Tukey test).

The most effective concentration (1000 ng/μl) of dsRNA was selected among all of the tested concentrations and fed to different aged (5 day, 7 day and 10 day) adult males for 6 hours. The sterility on 2^nd^ (new flies), 5^th^, 7^th^ and 10^th^ day-old males were noted as 85.40%, 31.42%, 21.76% and 9.90%, respectively compared to the control group (ds-EGFP) (Fig. 7).

### Number of Spermatozoa and live/dead sperm

**Figure 8:**
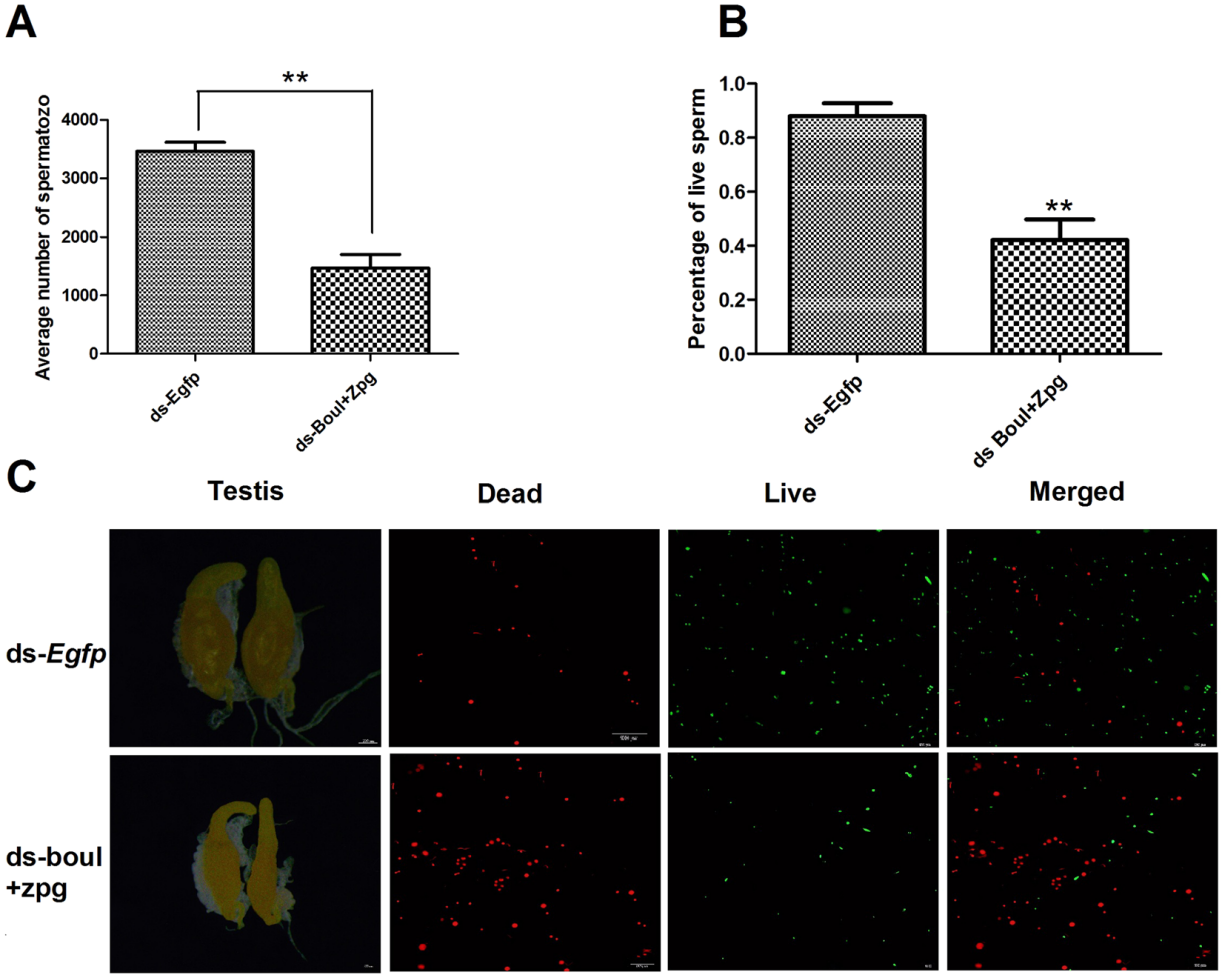
Average number of spermatozoa percentage of dead and live sperms. (**A**) Average number of spermatozoa in seminal vesicles, (**B**) percentage of live sperms per male was determined in seminal vesicles of 20 males per treatment, the effect of treatments was analyzed using T-test. ** indicates P < 0.01. (**C**) Microscopic picture of testis (200 μm) revealing the dead sperm (100 μm) (red) and live sperm (100 μm) (green).

The total number of spermatozoa were directly quantified in dsRNA-treated *B. dorsalis* males after 14 days and compared with the spermatozoa from the negative control, ds-EGFP -treated flies. Comparison with the control males revealed a significant reduction in the average number of sperm in seminal vesicles of ds-*boul* + ds-*zpg* treated male flies (Fig. 8A). Currently, it is uncertain whether the decline in the reproductive capacity of males (85.40%) is due to the reduction in number of spermatozoa. We hypothesized that the reduction in spermatozoa is not the only cause of the male sterility in flies. Therefore, we performed a sperm viability assay and found a significant difference between live and dead sperm in treated and control flies (Fig. 8B&C). The percentage number of spermatozoa and live sperm in treated flies was 57%, 52% reduced respectively as compared to control flies (P > 0.05).

## Discussion

Five testis-specific genes were selected in our study (*boul*, *Zpg*, *dsx* ^*M*^, *fzo* and *gas8*) and their efficacy was assessed by feeding genetically engineered-bacteria that express the target gene dsRNA. The results indicate that all of the examined genes severely impaired gene efficiency, but three of them (*boul*, *zpg* and *dsx* ^*M*^) depicted a significant effect on SIT in *B. dorsalis*. Moreover, to evaluate the synergistic effect of dsRNA, the most effective gene dsRNAs combinations were also prepared (*boul* + *zpg*, *boul* + *dsx* ^*M*^ and *zpg* + *dsx* ^*M*^) for pest management application by using SIT. With the use of dsRNA combinations, we achieved up to 85.40% male sterility. Our results help develop a new, environmentally friendly approach (SIT) based on RNAi/dsRNA to overcome the notorious pest *B. dorsalis*. Detailed laboratory experiments provided the appropriate and highly selective genes involved in spermatogenesis and showed significant results for the development of SIT.

Different gene dsRNAs were used through oral feeding, silenced the required genes, reduced the egg hatching and enhanced male sterilization in *B. dorsalis*. Due to the notorious nature of the model pest *B. dorsalis*, it has remained the focus for researchers. The feeding trial of dsRNA was previously established and reported from our lab^16, 17^ and it use in our present studies revealed that genetically engineered-bacteria can efficiently express target gene dsRNA and reduce the proficiency of relevant genes. However, sometimes the oral administration of dsRNA is not effective compared to other methods, such as micro-injection and topical application. This may be due to inappropriate concentrations, nucleotide sequence selection for siRNA application and environmental conditions of the target pest^18, 19^. However, in the current study, all of target gene dsRNAs displayed a significant silencing effect and egg hatching rates were highly reduced, except with the two genes, *fzo* and *gas8*. The lack of a significant reduction in egg hatching rate compared with the control may be due to an inappropriate concentration or to the development of resistance to the dsRNA^20^. Another dsRNA feeding trial was conducted with *Locusta migratoria* in response to different doses, ranging from 0.1 µg to 12 µg and it did not show a significant effect on the target gene *V-ATPase E*; however, a decline expression in mRNA was noted with the same genes at 18 µg^21^. Inappropriate concentrations of dsRNA can also cause the upregulation of target gene expression. A similar paradox of increased expression was noted in response to different concentrations of dsRNA^17, 22, 23^. It is very clear that proper nucleotide sequence, proper concentration and accurate modes of application are necessary to determine the exact silencing effects of dsRNA^20^.

Our current results demonstrated an increase in the silencing effect and in male sterility in the target pest in response to target gene dsRNA (Fig. 5). Similar studies were conducted and revealed the effectiveness of boul gene dsRNA in male sterility against *Bactrocera oleae*. They revealed that males of the lead strain, OX3097D-*Bol*, 1) are strongly sexually competitive with wild olive flies, 2) display synchronous mating activity with wild females, and 3) induce appropriate refractoriness to wild female re-mating. Furthermore, they showed, through a large proof-of-principle experiment, that weekly releases of OX3097﻿D-*Bol* males into stable populations of caged wild-type olive fly could cause rapid population collapse and eventual eradication﻿^3^. Our results display greater efficiency and we found a significant reduction in spermatozoa (Fig. 8A). Our findings confirm that the selection of target genes is very important. It could be argued that a mutation of the *boul* gene in *Drosophila* and human could cause deletion of azoospermia (production of no sperm). The production of no sperm has also been explained by a blockage of meiosis during cell division^24, 25^. The deletion of azoospermia is associated with a significant change in testicular features, which leads to the absence of germ line cells involved in spermatogenesis^26^. In addition, *zpg* encodes for the germline gap junction protein innexin4, which plays a very crucial role in gametogenesis, especially during early differentiation of the germ cell^15^. Mutations in *zpg* caused sterility and smaller gonad production^15^. Recent research in *D. melanogaster* demonstrated that the gap-junction is the direct or indirect pathway between the germline and the soma. When any disturbance occurred in the gap junction, it ultimately blocked the GSC and led to insect sterility^27^. The significant difference between live and dead sperm (Fig. 8B,C) also confirms the maintenance of the stem cell, as nourishment of sperm and testis are directly controlled by gap junction-derived cues^27^. *Dsx* ^*M*^ also plays a very important role in the development of gonad cells during formation of the testis^14^. Ma *et al*.^28^ revealed that the canonical male sex expression factor, “*dsx* ^*M*^” is promoted by chinmo and it also prevents the feminization of somatic cyst stem cells (CySCs).

RNAi is an advanced and powerful molecular tool to overcome the pest population. The use of SIT based on RNAi is more effective than the radiation at sterilization because it is environmentally safe, retains the male fitness, directly affects a specific sequence and reduces gene functions, such as reproduction ability. Our results showed that the *boul*, *zpg* and *dsx* ^*M*^ gene dsRNAs caused a high percentage of sterility and examination of their combinations revealed that *boul* + *zpg* is the most effective combination for producing sterile males. Our sequence based target genes are present in other species and it may open a new industry for pest control.

From our research, we could not determine the exact cause of male sterility or roles of the target genes in spermatogenesis. In addition, numerous barriers exist for the commercial use of this technique, including attainment of 100% sterility, proper timing of dsRNA application, concentration, fragment length and persistence.

## Materials and Methods

### Insect Rearing

Adults of *B. dorsalis* were kept in 28 cm × 28 cm × 28 cm cages^17^ and provided with a constant supply of double distilled water and an artificial diet containing 7.5% sugar, 2.5% yeast extract, 0.5% agar 2.5% honey and 87% water^29^. Oviposition was carried out in yellow plastic containers in 3-cm petri plates with banana pulp. The larvae were nourished as previously reported^30^. Adults and larvae were kept at 28 ± 1 °C, with relative humidity 75 ± 5% and on a 12 L:12 D photoperiod cycle.

### Selection of Target genes

For RNAi, 10 genes were initially selected based on previous studies (Table S1). Genes Specific primers were designed by using the NCBI-Primer-BLAST **(** https://www.ncbi.nlm.nih.gov/tools/primer-blast/ **)** database and quantitative real-time PCR (qRT-PCR) analysis was performed to evaluate gene expression in different body parts. For SIT, genes with high expressions in the testis were selected for further experiments.

### Cloning and sequencing

Total RNA was isolated from fully emerged male adult insects using TRIzol reagent (Invitrogen). First strand cDNA was synthesized with the help of a commercially available Kit (ThermoScientific, USA) following the manufacturer’s genes (*boul, zpg, dsx* ^*M*^ *, fzo*, & *gas8*) primers were designed by using the NCBI- instructions. Five target Primer-BLAST (**(** https://www.ncbi.nlm.nih.gov/tools/primer-blast/ **)**) based on available transcriptomic data from *B. dorsalis* ^31^. The PCR products were run on agarose gel electrophoresis and the resultant bands were purified by Gel Extraction Kit (Omega, USA). For digestion, two restrictions enzymes (HindIII & Sac1) were used and purified digested products were ligated into the plasmid L4440. Sequencing analysis was performed on the resultant recombinant plasmids (Invitrogen, shanghai, China).

### Targeted dsRNA Expression

The expression of dsRNA was induced using L4440 plasmids in the HT115 (DE3) cell strain. The targeted genes were removed from the cloning vector using SacI and HindIII digestion and ligated into a similarly digested L4440 vector using T4 DNA Ligase (TaKaRa, China). The *Escherichia coli* HT115 competent cells were transformed with recombinant vectors (L4440-*boul, zpg, dsx* ^*M*^ *, fzo*, & *gas8*) and the culture was spread on plates with solid Luria Broth (LB) media with 100 μg/ml ampicillin. Single colonies of HT115 were picked and cultured in liquid LB media while shaking at 220 rpm overnight at 37 °C. The culture was diluted 100-fold in 800 ml if YT supplemented with 80 mg/ml ampicillin, 12 mg/ml tetracycline cultured at 37 °C and 0.6 optical density 600. Target genes dsRNAs were synthesized by following a previously determined protocol^29^. In brief T7 polymerase synthesis was induced with 0.4 mM IPTG and the bacteria were incubated at 37 °C for additional 5 h on shaking. According to Timmons^32^ HT115 bacteria solutions were centrifuged for 10 min at 5,000 g and to condense the concentration to 500 × re-suspended in 1 M ammonium acetate then incubated at 65 °C for 20 minutes while adding the phenol, chloroform and isoamyl alcohol (25:24:1). Right after, centrifugation was done at 15,000 g for 10 minutes and upper phase was separated into clean tube with already containing same volume of isopropanol and store at 20 C for overnight. The highly concentrated nucleic acid pellets were obtained after the centrifugation at 15,000 g for 30 minutes. Nucleic acids were treated with RNase A solution (Promega), DNase (Promega) and RQ1 RNase-free, after being resuspended in DEPC-treated H_2_O. The quality of the dsRNA was analyzed on a 1.5% agarose gel stained with ethidium bromide and the concentration was calculated using Nano-Drop 1000 (ThermoScientific).

### dsRNA feeding

Feeding of dsRNA was conducted as described by Li *et al*.^18^. Newly emerged males and females were separated into individual rearing containers (17 cm × 8 cm × 8 cm), with each treatment having ~100 males or females. Male flies were starved/ dehydrated for 24 h before feeding them the dsRNA. The artificial diet was covered with 1 ml of dsRNA (1 µg/µl) on plates. In addition, dsRNA of green fluorescent protein (ds-EGFP) was used as the control. After 6 hours of dsRNA feeding, all insects were transferred to a normal diet, as discussed above. Target gene dsRNA treated adults were allowed to mature on an artificial diet until they were 12 days old. Immediately after sexual maturation, virgin females of the same age were allowed to mate with males and examined for egg laying and hatching capacity.

### Quantitative real-time PCR (qPCR) Analysis

Total RNA was extracted from 15 flies every 24 hours. First strand cDNA was synthesized as mentioned above. Real time PCR analysis was performed by using iTaq™ Universal SYBR Green Supermix (BioRad) on a Bio Rad iCycler by following the manufacturer’s instructions. 16 S rRNA was used as internal gene control^17^. The thermal cycler conditions were maintained according to previously reported in our lab^29^. All the analyses were repeated in triplicates. qPCR analysis was performed by the 2−ΔΔCT method, as described by Livak and Schmittgen^33^. All the primers for qPCR analysis were designed by NCBI database from the ORF (Table S3) to avoid the same portion used for dsRNA synthesis^31^.

### Reproduction bioassays

After 13 days of feeding trials on a normal artificial diet, 20 pairs of virgin males and females were selected and reared in a new cage for mating, as discussed above. After 24 hours of mating, eggs were collected after 25 minutes of being placed on the banana-containing, yellow plastic cup. The eggs were gently collected, placed on black filter paper (A4) and counted manually. Subsequently, for hatching efficiency, eggs were placed on banana pulp in a controlled environment. After 3–5 days, hatched larvae were counted and the reproductive efficiency was calculated in percentage from online calculator (http://marshu.com/articles/calculate-percentage-increase-decrease-percent-calculator.php) as compared to control.

### Determining technique factors for the SIT (Individual and synergistic effects of dsRNA on male sterility)

Individual and synergistic effects of different gene dsRNAs on male fertility was also explored. Experiments were carried out by using the most effective gene combination (*boul* + *zpg*) at the most effective concentrations and optimal adult stages to determine technique factors for the SIT. In this regard new adult male flies were allowed to feed on different concentrations (250, 500, 750 and 1000 ng/μl) of dsRNA for 6 hours. Before the feeding of dsRNA, flies were starved and dehydrated for 24 hours. After six hours of feeding, all flies were shifted to a normal artificial diet until they were sexually mature. The13-day-old flies were allowed to mate with same aged virgin females. The eggs were counted by following the previously described procedure. Immediately after, the best concentration was selected and another male fertility assay was performed by feeding the different aged (5, 7 and 10 days) adult males in response to the target dsRNA concentration (1000 ng/μl).

### Sperm viability assays and spermatozoa counts

Dissection of seminal vesicles of each insect was performed in Hayes solution (9 g of NaCl, 0.2 g of CaCl_2_, 0.2 g of KCl and 0.1 g of NaHCO_3_ in 1,000 ml of H_2_O) and watchmaker forceps were used carefully to puncture the seminal vesicles. With the help of a pipette, an out-flowing sample of 2 μl of sperm was collected and further diluted in Hayes solution (250 μl). Using a previously established protocol, the Live/Dead™ sperm viability kit (L-7011, Molecular Probes) was used to measure sperm viability. The kit comprises of a membrane-permanent nucleic acid stain for live sperm (SYBR-14) showing green emission and a dead cell stain (propidium iodide) displaying red emission^16, 34, 35^. For each measurement, incubation was carried out for 10 min at room temperature by adding 5 μl of a SYBR-14 working solution (2 μl of SYBR-14 stock in 98 μl of Hayes saline) to 5 μl of the sperm samples on a microscope slide. Subsequently, in each sample 2 μl of propidium iodide was added and incubated for 7 min at 25 °C in a dark humid box to inhibit desiccation. Afterward, by using a fluorescence microscope (Olympus CX41, EXFO X-Cite 120, filter cube CX-DMB-2, x400–800 magnification), the number of dual-stained (both green and red), live and dead sperms were counted carefully from 400 randomly selected sperm cells per sample. From the total sperm population, dual stained cells (maximum of 1.6% per sample) were discarded from the data. The live sperm percentage in the total number of sperm was counted and sperm viability was calculated for each sample. In addition, sperm were killed by freezing at −80 °C for 8 h to validate the experimental protocol. As predicted, all sperm sample stained red (dead). The method described by C. Bressac and C. Chevrier was used to count sperm^36^. In short, sperm were extracted, followed by the fixation of spermatozoa with the use of ethanol, air-dried and stained with DAPI for 15 minutes. Then, using a fluorescence microscope, individual spermatozoa nuclei were visualized and counted.

### Data analyses

The data were statistically analyzed using Tukey’s test and One-way analysis of variance (ANOVA) at P < 0.0001 by using GraphPad prism 5.0 and represented as the mean ± SE. An independent samples t-test was carried out for comparing the hatching rate of eggs per day, accumulative normalized eggs hatching and proportion of number of eggs lay per day.

## Electronic supplementary material

Supplementary Data
