# Supplementary material for: A genetically enhanced sterile insect technique against the fruit fly, Bactrocera dorsalis (Hendel) by feeding adult double-stranded RNAs
Source: Sci Rep. 2017 Jun 22;7:4063. doi: 10.1038/s41598-017-04431-z (PMC5481416; doi:10.1038/s41598-017-04431-z)
Supplement: Supplementary file 1 — Supplementary Data [file 41598_2017_4431_MOESM1_ESM.pdf]

A genetically enhanced sterile insect technique against the fruit fly, *Bactrocera dorsalis* (Hendel) by feeding adult double-stranded RNAs

Muhammad Waqar Ali [1], Wenping Zheng [1], Summar Sohail [1], Qingmei Li [1], Weiwei Zheng [1], Hongyu Zhang [1,]*

[1] Key Laboratory of Horticultural Plant Biology (MOE), State Key Laboratory of Agricultural Microbiology, Institute of Urban and Horticultural Entomology, College of Plant Science and Technology, Huazhong Agricultural University, Wuhan 430070, China. Correspondence and requests for materials should be addressed to H.Z. (Email: hongyu.zhang@mail.hzau.edu.cn)

Supplementary Tables

Table S1 Genes expressed predominantly in testis and ovary of *Bactrocera dorsalis*

***Bactrocera dorsalis* accession#**	***D. melanogaster* homology**	**Testis-specific**	**Ovary Specific**
XP_011211203.1	CG8208 (MDB like)	No	Yes
XP_011212712.1	CG4727(Boule)	yes	No
XP_011213454.1	CG14271 (Gas8)	yes	No
XP_011201483.1	CG4568 (fzo)	yes	No
XP_011210342.1	CG5737 (dmrt93B)	No	No
XP_011213617.1	CG6647 (*Zpg*)	yes	Yes
XP_011200249.1	CG12423 (klhl10)	No	Yes
XP_011207287.1	CG2146 (diddum)	No	Yes
XP_011207287.1	CG12813 (Npc2d)	No	No
XP_011203913.1	NP_001262353.1 (dsx^M)	yes	No

Table S2 Primers used to verify the expression of target genes in different body parts by qRT-PCR analysis

Genes	Sequence	Gene expression in different body parts
(XP_011211203.1) *MDB like* F	AAGCGCAAATTCAAAGCGCA	q-Real-time PCR
(XP_011211203.1) *MDB like* R	GGGGCACAACCGCTACTAAT	q-Real-time PCR
(XP_011212712.1) *Boul* F	CTGTTTGGTATAAGCGTGCCA	q-Real-time PCR
(XP_011212712.1) *Boul* R	CAACCACAGCCAATGCACAA	q-Real-time PCR
(XP_011213454.1) *Gas8* F	GATGATCTGCTCGCCACGTA	q-Real-time PCR
(XP_011213454.1) *Gas8* R	GCTGGGCCGCATAAATAACG	q-Real-time PCR
(XP_011201483.1) *Fzo* F	CTGCACGCAGAAGCTGAAAT	q-Real-time PCR
(XP_011201483.1) *Fzo* R	GAGTACCTCACGTATCGCGG	q-Real-time PCR
(XP_011210342.1) *dmrt93B* F	AATCGGTATGGGTGTATGGGT	q-Real-time PCR
(XP_011210342.1) *dmrt93B* R	ATTGACTTTGGCGTCTCGCAT	q-Real-time PCR
(XP_011213617.1) *Zpg* F	ACAGCAAATTCAAACCGGCG	q-Real-time PCR
(XP_011213617.1) *Zpg* R	GCGCGATGTGCTTTGTATTCT	q-Real-time PCR
(XP_011200249.1) *klhl10* F	AGGTTAAGCGTTGGCTTGATA	q-Real-time PCR
(XP_011200249.1) *klhl10* R	TGTGTGGACACATTGCGAAG	q-Real-time PCR
(XP_011207287.1) *diddum* F	TTTGCTGCAGTTGGAGGTTCT	q-Real-time PCR
(XP_011207287.1) *diddum* R	TTTGCATTGCCGAATGCCTC	q-Real-time PCR

Table S2(Continued)

Genes	Sequence	Gene expression in different body parts
(XP_011207287.1) *Npc2d* F	CTTTCGAGCCGGTCAGGTAG	q-Real-time PCR
(XP_011207287.1) *Npc2d* R	ACCGTCGACGCTGTATGAAG	q-Real-time PCR
(XP_011203913.1) dsx^M F	ACAGCGACACAATGTCCGAT	q-Real-time PCR
(XP_011203913.1) dsx^M R	GTGCACAATTGGGTGGAGTG	q-Real-time PCR

Table S3 Primers used to amplify target gene fragments for dsRNA synthesis and for qRT-PCR analysis

Primers	Sequence	Purpose
Boul F	AGCGCCGAAAAAGCTCAAAG	Gene cloning
Boul R	CTGTGGCCAAATGGTGGGTA	Gene cloning
ds-*Boul* F	CCCAAGCTTTATCCGCCCCAAAGTATGGC	dsRNA synthesis
ds-*Boul* R	CGAGCTCACCGTAACCCTTGCTGACTC	dsRNA synthesis
Q *Boul* F	GCCGGCATGCCAACAATTTA	q-Real-time PCR
Q *Boul* R	TGGCCAAATGGTGGGTACAT	q-Real-time PCR
Zpg F	ACAAAAATTTGCGATTTCGTGTTTG	Gene cloning
Zpg R	CAGCAAATTCAAACCGGCGA	Gene cloning
ds-Zpg F	CCCAAGCTTCGGTGAAACCGCTCTCCAAA	dsRNA synthesis
ds-*Zpg* R	CGAGCTCTTGTCATCGCCCAGACATTG	dsRNA synthesis
Q *Zpg* F	CAAGCGCGATGTGCTTTGTA	q-Real-time PCR
Q *Zpg* R	CAGCAAATTCAAACCGGCGA	q-Real-time PCR
Dsx^{M} F	CGTAGAGGCCTCGCTTTTGT	Gene cloning
Dsx^{M} R	TTTCCTAGGCTTCCGGGACA	Gene cloning
ds-Dsx^{M} F	CCCAAGCTTTCCGATTGCCATTGGAAGCA	dsRNA synthesis
ds-Dsx^{M} R	CGAGCTCGGCGTACGACTTAGAGTGGG	dsRNA synthesis
Q *Dsx*^{M} F	CATCTAAGCCTTCGCGACCA	q-Real-time PCR
Q *Dsx*^{M} R	ACGGATGACGGCAAGTTTGT	q-Real-time PCR

Table S3 (Continued)

**Primers**	**Sequence**	**Purpose**
Fzo F	GCGAAAGACGCGCCTAAAAT	Gene cloning
Fzo R	AGCCTGAAGAGTTTCAAGTG	Gene cloning
ds-Fzo F	CCCAAGCTTAACGCTAATGCAAACGCCG	dsRNA synthesis
ds-*Fzo* R	CGAGCTCGCTTCTGCGTGCAGAGCATT	dsRNA synthesis
Q *Fzo* F	TGTACATAAAGTGGCCGCGA	q-Real-time PCR
Q *Fzo* R	CTCTTGCCATTCGATGTGCG	q-Real-time PCR
Gas8 F	GAGCAAAAGCACGAACCTCG	Gene cloning
Gas8 R	TACGCACACAGGCTCGTATC	Gene cloning
ds-Gas8 F	CCCAAGCTTGCAAGAAGGCGCCAATGTTA	dsRNA synthesis
ds-*Gas8* R	CGAGCTCAGTTCATGCGTTTCGTCGTG	dsRNA synthesis
Q *Gas8* F	AAAGTCACGACACCGCCTTC	q-Real-time PCR
Q *Gas8* R	GCGCTTCGAGTTGTTCCTTC	q-Real-time PCR
Q 16s F	CTCGTCCAACCGTTCATACC	q-Real-time PCR
Q 16s R	CTGACCTGCCCACTGAAGTT	q-Real-time PCR
